# *In Silico* Characterization of Uncharacterized Proteins From Multiple Strains of *Clostridium Difficile*

**DOI:** 10.3389/fgene.2022.878012

**Published:** 2022-08-11

**Authors:** Bilal Ahmed Abbasi, Aishwarya Dharan, Astha Mishra, Devansh Saraf, Irsad Ahamad, Prashanth Suravajhala, Jayaraman Valadi

**Affiliations:** ^1^ Bioclues.org, Hyderabad, India; ^2^ Amrita School of Biotechnology, Amrita Vishwa Vidyapeetham, Clappana, India; ^3^ School of Computational and Data Sciences, Vidyashilp University, Bengaluru, India; ^4^ Department of Computer Science, FLAME University, Pune, India

**Keywords:** clostridium difficile, uncharacterized proteins, essential genes, annotation, function abbreviations C. difficile-clostridium difficile CDI-C. *difficile* infection

## Abstract

*Clostridium difficile* (*C. difficile*) is a multi-strain, spore-forming, Gram-positive, opportunistic enteropathogen bacteria, majorly associated with nosocomial infections, resulting in severe diarrhoea and colon inflammation. Several antibiotics including penicillin, tetracycline, and clindamycin have been employed to control *C. difficile* infection, but studies have suggested that injudicious use of antibiotics has led to the development of resistance in *C. difficile* strains. However, many proteins from its genome are still considered uncharacterized proteins that might serve crucial functions and assist in the biological understanding of the organism. In this study, we aimed to annotate and characterise the 6 *C. difficile* strains using *in silico* approaches. We first analysed the complete genome of 6 *C. difficile* strains using standardised approaches and analysed hypothetical proteins (HPs) employing various bioinformatics approaches coalescing, including identifying contigs, coding sequences, phage sequences, CRISPR-Cas9 systems, antimicrobial resistance determination, membrane helices, instability index, secretory nature, conserved domain, and vaccine target properties like comparative homology analysis, allergenicity, antigenicity determination along with structure prediction and binding-site analysis. This study provides crucial supporting information about the functional characterization of the HPs involved in the pathophysiology of the disease. Moreover, this information also aims to assist in mechanisms associated with bacterial pathogenesis and further design candidate inhibitors and *bona fide* pharmaceutical targets.

## Introduction

*Clostridium difficile* is a multi-strain, spore-forming, Gram-positive anaerobic bacterium posing a global threat to post-operative individuals. Infamously known for antibiotic-associated diarrhoea, it is one of the most important causes of healthcare-associated infections worldwide, leading to a quarter of reported cases of infectious diarrhoea and a broad spectrum of gastrointestinal complications, including sepsis and pseudomembranous colitis (Barbut and Petit, 2001). Studies have suggested that it is a crucial part of healthy human gut flora as it overgrows and imbalances intestinal microflora with unnecessary antibiotic therapies (Abt et al., 2016). With the progression of antibiotic-based therapeutics accompanied by sub-standard hygiene in hospitals, the incidence of *C. difficile* infection (CDI) has significantly increased since the 20th century (Czepiel et al., 2019). Being a major causative pathogen, *C. difficile* contributes to almost half a million cases with 29,000 deaths per annum in the United States alone and impacting Latin America, Europe, and the Asian regions (Goudarzi et al., 2014; Lessa et al., 2015). Whereas in India, the incidence and prevalence rates of CDI-associated diarrhoea in hospitalised patients ranges from 3 to 29% and 7.1–26.6%, respectively (Segar et al., 2017).

*C. difficile* possesses a huge, diversified pangenome with high levels of evolutionary plasticity accumulated over time due to gene flux and recombination in response to environmental changes. Literature suggests the evolutionary rate of *C. difficile* to be 3.2 × 10^–7^ mutations per nucleotide per year, resulting around 1.4 mutations per genome per year that drives and reshapes the genetic diversity of the pathogen (Didelot et al., 2012). Additionally, the ratio of the nucleotide substitution rate to result of mutation (r/m) has been estimated around 0.2 or higher. These rates are comparatively lower to other guts pathogens (He et al., 2010). *C. difficile* infection involves an opportunistic colonisation of the intestinal tract leading to nosocomial, antibiotic-associated severe diarrhoea with or without colitis, fever with chills, and abdominal pain (Bartlett, 2002; Korman, 2015). *C. difficile* infection occurs via transmission of spores that are resistant to acid, heat and antibiotics. Antibiotics like metronidazole and oral vancomycin have been recommended as a cure for the acute infection. Other antibiotics including penicillin, tetracycline, and clindamycin, have been employed to control CDI, but studies have suggested that imprudent overuse has led to the development of antimicrobial resistance in *C. difficile* strains (Nelson et al., 1994). Current treatments for CDI consist of supportive care, discontinuation of unnecessary antibiotics and specific antimicrobial therapies.

Furthermore, novel methodologies including fidaxomicin therapy, and faecal microbiota transplantation-mediated therapy have shown prominent results. Faecal microbiota transplantation has shown significant efficacy to overcome CDI and reduce its recurrence (Goudarzi et al., 2014). The appearance of hyper-virulent antibiotic-resistant strains with the production of antimicrobial peptides from activated immune cells and inflamed epithelial cells allows the residual *C. difficile* to re-expand, following the end of treatment (Vindigni and Surawicz, 2015). Growth and development of the bacteria can be prevented at the genetic level effectively, by reducing the prevalence of *C. difficile* and also limiting the rates of recurrence (Leber et al., 2017). The pathophysiology of the disease, including the transmission and physicochemical pathways employed by *C. difficile,* has aroused several researchers in the past few years to investigate the proteins involved in their virulence (Rineh et al., 2014; Smits et al., 2016).

Advanced high-throughput technologies like genome sequencing, gene editing, and functional annotation might be helpful to understand the biology of *C. difficile* and its genomic composition. In the recent past, domains like genomics, transcriptomics and proteomics studies have assisted in gaining insights to the mechanism of microbial adaptation (Sebaihia et al., 2006; Stabler et al., 2009; Boetzkes et al., 2012; Cafardi et al., 2013). Moreover, there are still challenges while decoding these mechanisms, and the bioinformatics tool aids in our understanding via functional annotation, protein-protein interactions, and pathway analysis. Functional annotation of uncharacterized proteins is a crucial step in deciphering the role of proteins. An uncharacterized or hypothetical protein (HP) is defined as the one that is predicted to be expressed in an organism but no proper function is known (Suravajhala et al., 2015). Most of these hypothetical proteins are expected to play essential roles and their annotation can unveil novel functional pathways. The utilisation of *in silico* approaches to predict annotations of HPs has been successful in numerous bacterial species (Singh et al., 2015; Varma et al., 2015; Prabhu et al., 2020). Additionally, there are still several challenges in annotating such proteins, given the scanty organelle information known and the pervasive nature of the subcellular location of these proteins. Earlier, methods to annotate HPs by us (Ijaq et al., 2019) could be useful, but given the bacterial system, a coherent need for employing several computational tools, viz. determine the conserved domain, subcellular localization, secretory nature, physicochemical characterization, identification of prophage sequence and CRISPR-Cas9 system, detection of antimicrobial resistance, comparative homology analysis, virulence factors, antigenicity analysis, allergenicity determination along with structure prediction and binding-site analysis would allow us to annotate the possible functions for the HPs. Deciphering the role of complete gene coding regions in the genome is crucial to merge the gaps in the proteome to fully understand the pathogenicity. This study aims to determine the functional annotations of HPs of *C. difficile* to have a clear implication.

## Materials and Methodology

### Data Retrieval

**FIGURE 1:**
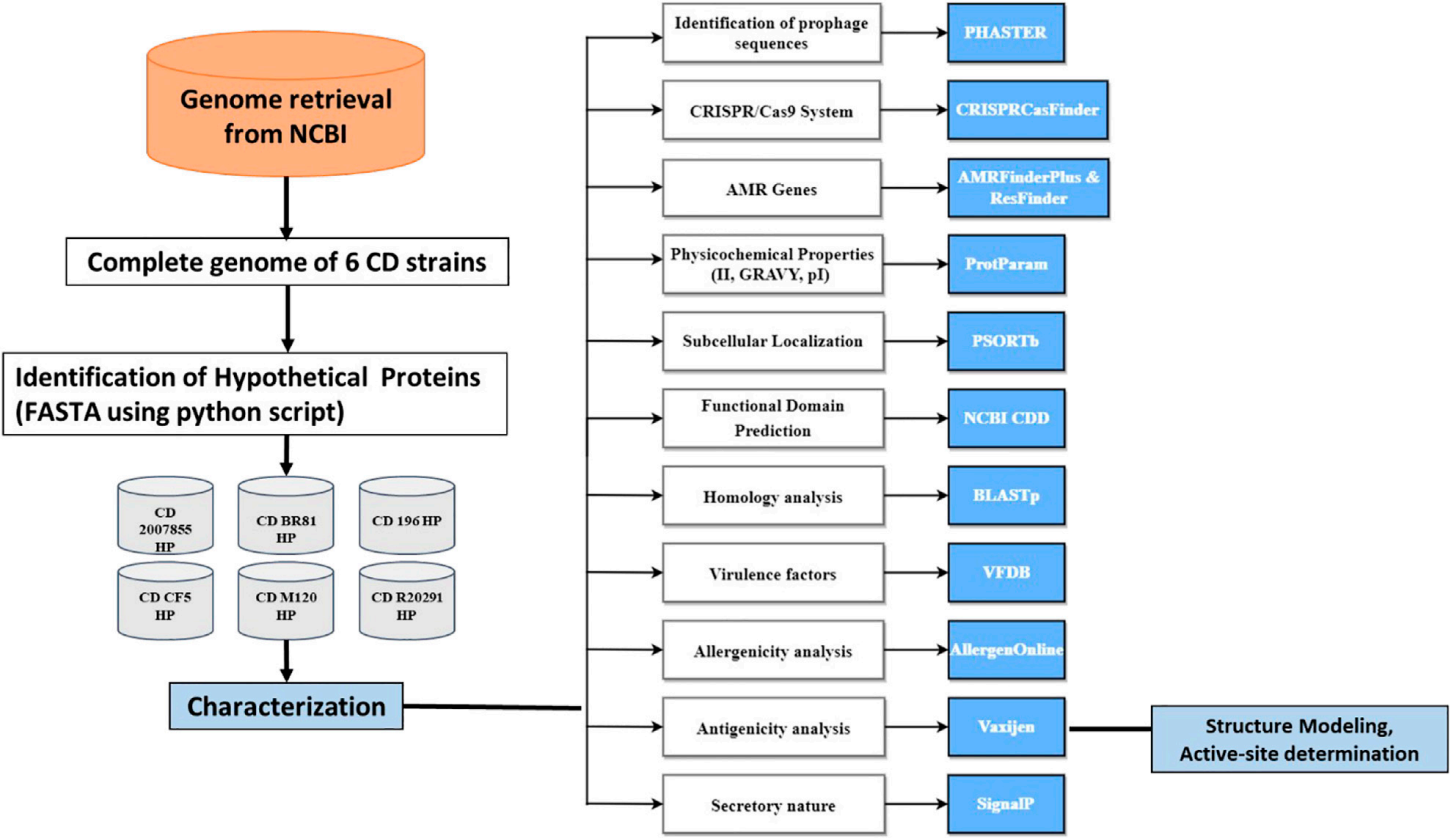
Methodology used for characterization of HPs for *Clostridium* strains. Briefly, we have jotted down the HPs using python script and characterised the six strains of *C. difficile* using a cohort of tools (Please see methodology). The subsequent annotation is tabulated and analysed.

A total of 2,512 genomes of *C. difficile* were available in the NCBI database (25 Aug 2021). A robust methodology was used to narrow down these 2,512 genomes to retrieve the complete genome of six strains of *C. difficile*, namely, BR81, R20291, CF5, M120, 196, and 2,007,855. Initially, this obtained data was standardised using RAST pipeline, which is an automated service that gives high-quality genome annotations for complete or nearly complete bacterial and archaeal genomes (Overbeek et al., 2014). Finally, HPs were extracted from proteomes of these *C. difficile* strains using a python script. The protocol used in this study is depicted (Figure 1).

### Identification of Prophage and CRISPR-Cas9 System

CRISPR-Cas9 system acts as an adaptive immune system in microbes against prophages. It uses RNA guided nucleases to cleave foreign genetic elements (Ran et al., 2013). It is also responsible for a continuous saga of evolution between phages and bacteria via addition or deletion of spacers into the genome of host bacteria and via mutations or deletion in phage genomes (Deveau et al., 2010). Thus, prophage aids in understanding the evolution of bacterial genomes. The PHASTER server was employed for the identification of prophage sequences in the whole genomes of bacterial strains (Arndt et al., 2016). It queries virus and prophage/bacterial databases to identify potent prophage sequences and rank the hits according to score; as intact (score>90), questionable (score ranging between 70 and 90), or incomplete (score<70). Additionally, CRISPRCasFinder was employed to identify CRISPR-Cas9 related genes in six strains of Closteroides using the default settings (Couvin et al., 2018).

### Identification of Antimicrobial Resistance

In order to identify AMR genes, two different procedures were utilised. Firstly, the identified HPs from the six CD strains, and RAST based characterization of WGS were searched for the presence of AMR genes using the tools AMRFinderPlus v3.10 and ResFinder v 4.1 (Bortolaia et al., 2020; Feldgarden et al., 2021). Default settings with a maximum coverage length of 80% and percent identity set at 90% were used for AMRFinder Plus. Default settings with % ID threshold set at 80% were used for ResFinder.

### Physicochemical Characterization of Hypothetical Proteins

ExPASy’s ProtParam tool evaluated various physicochemical properties for the obtained HPs. The ProtParam tool determines these properties based on the amino-acid sequence. For the present study, we computed properties like theoretical isoelectric point (pI), molecular weight, instability index, and grand average of hydropathicity (GRAVY) value. The instability index estimates whether a protein will be stable in the test tube or not. Proteins having an instability index lesser than 40 are predicted to be stable, whereas a value greater than 40 indicates the protein to be unstable. A negative GRAVY value implies the protein is non-polar, while a positive value means the protein is polar (Gasteiger et al., 2005).

### Identification of Subcellular Localization and Secretory Nature

Each bacterial protein is localised into different subcellular locations like cytoplasm, plasma membrane, outer membrane etc*.* and can perform different functions. Thus, subcellular localization is a chief criterion for identification of potential bacterial drug targets (Omeershffudin and Kumar, 2019). PSORTb 3.0 tool was used for assigning the subcellular localization of HPs (Yu et al., 2010). The tool utilises a support vector machine that gives scores related to subcellular classifiers of each protein based on their amino acid sequences and evaluates their probability of finding the final location. Additionally, another tool, SignalP 5.0 server was used to predict whether the HPs from 6 *C. difficile* strains are secretory or non-secretory proteins in nature. The server predicts the presence of signal peptides and the location of their cleavage sites in proteins. In bacteria, it can discriminate between three signal peptides, Sec/SPI, Sec/SPII, and Tat/SPI, based on how they are transported and cleaved (Petersen et al., 2011).

### Functional Domain Prediction

NCBI Conserved Domain Search Service (CDD) was implemented to investigate the domains of the selected HPs. It identifies the conserved domains present in protein sequences by performing Reverse Position Specific (RPS)-BLAST against position specific scoring matrix (PSSM) resulting from conserved domain alignments present in the conserved domain database (Marchler-Bauer et al., 2015).

### Comparative Homology Analysis

The homology analysis of HPs against the human proteome was performed using the BLASTp tool. The proteins with ≥35% identity, ≥35% query coverage, and <10e-5E value were considered homologous to human proteins. The hypothetical non-homologous protein can be used to design potential vaccine candidates against *C. difficile* since those will avoid generating potential cross-reactivity (Altschul et al., 1990).

### Prediction of Virulence Factors

Bacterial virulence factors are the molecules, cell structures, or regulatory pathways that allow the microbes to replicate and spread within the host by evading or suppressing the host’s immune response. These can serve as targets for identifying new therapies against the disease. The Virulence Factor Database (VFDB) was used for determining whether the identified HPs are virulent factors or not (Chen et al., 2005).

### Antigenicity Analysis

Identification of novel antigens associated with infectious diseases are essential for invention of new diagnostic tests as well as designing subunit vaccines against them (Liang and Felgner, 2012). Thus, it is important to identify if the HPs from six strains of *C. difficile* are antigenic in nature. To predict the antigenicity of the HP, an online server, Vaxijen was used with the default settings for Gram positive bacteria (Doytchinova and Flower, 2007).

### Structure Prediction and Active Site Determination

With the results of the previous step, the highest antigenic proteins were identified and further subjected to structure modelling via iTASSER structure prediction server (Roy et al., 2010). These three-dimensional proteins were further employed and investigated for active site determination using CastP server (Tian et al., 2018).

### Allergenicity Analysis

AllergenOnline database was used to predict whether the HPs are allergic to humans in nature. This information helps in determining whether the HPs are potentially allergenic. Non-allergenic proteins can be utilised for designing vaccine candidates (Goodman et al., 2016).

## Results

### Data Retrieval

**FIGURE 2:**
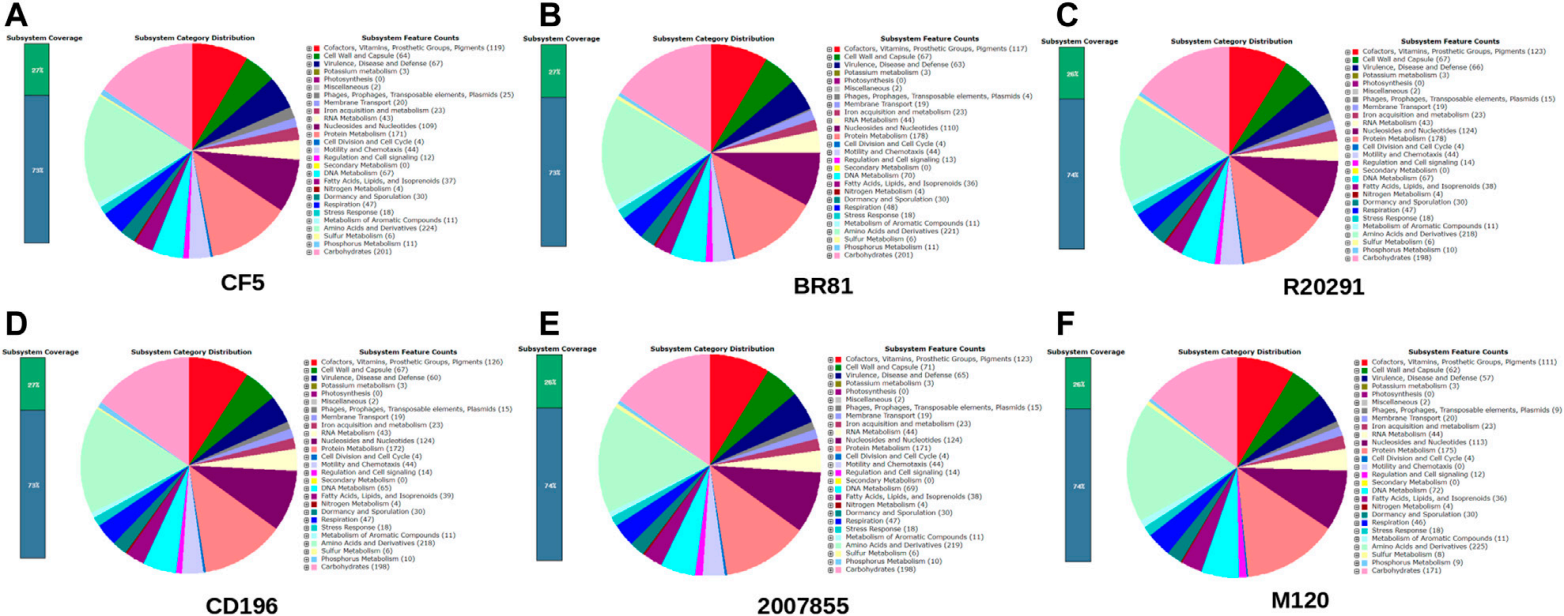
Overview of Subsystem Category Distribution for six strains **(A)** *C. difficile* strain CF5. **(B)** *C. difficile* strain BR81 **(C)** *C. difficile* strain R20291 **(D)** *C. difficile* strain 196 **(E)** *C. difficile* strain 2,007,855 **(F)** *C. difficile* strain M120. Identification of Prophage and CRISPR-Cas9 systems.

In this study, six complete genomes from *C. difficile* were utilised. All the genomes were retrieved from the NCBI database (https://www.ncbi.nlm.nih.gov/genome/) and standardised using RAST (Overbeek et al., 2014). The key characteristics of the strains used in this study are listed here (Table 1 and Figure 2).

**TABLE 1:** Characteristics of selected genomes of *Clostriodioles* strains.

S. No	Description	Accession ID	Size (Mb)	Proteins (HP)	GC%	CDS
01	*C. difficile* BR81	CP019870.1	4,124,384	3,547 (356)	28.7	3,683
02	*C. difficile* R20291	NZ_CP029423.1	4,204,902	3,647 (409)	28.9	3,802
03	*C. difficile* CF5	NC_017,173.1	4,159,517	3,587 (413)	28.5	3,797
04	*C. difficile* M120	FN665653.1	4,047,729	3,446 (404)	28.7	3,697
05	*C. difficile* 196	NC_013,315.1	4,110,554	3,552 (374)	28.6	3,715
06	*C. difficile* 2,007,855	NC_017,178.1	4,179,867	3,614 (377)	28.7	3,806

### Identification of Prophage and CRISPR-Cas9 System

The PHASTER tool was employed to identify the phage genes, if any, present in six strains of *C. difficile*. Four Intact Phage sequences (score>90) were found in *C. difficile* strain of R20291, CF5, 196, and 2,007,855, the details of which are provided here (Table 2). CRISPRCasFinder was employed to identify any CRISPR/Cas system located in the 6 *C. difficile* strains. The significance level of the predicted systems was evaluated based on the evidence level. Of all the *C*. *difficile* selected, the majority of the sequences had more than one spacer sequence and an Evidence level of 3-4, which suggests the presence of CRISPR/Cas genes (Supplementary Table S1).

**TABLE 2:** Intact Prophage region identified in *Clostriodium difficile* strains.

Genome	Intact Region	Region length (Kb)	Score	Total protein	Position	Common phage	GC%
*C. difficile* R20291	1	55.9	140	71	1,684,408–1,740,383	PHAGE_Clostr_phiMMP01_NC_028,883 (32)	28.64
*C. difficile* CF5	1	56.2	130	74	1,707,633–1,763,916	PHAGE_Clostr_phiMMP03_NC_028,959 (30)	29.02
*C. difficile* 196	1	57.7	140	72	1,673,218–1,730,955	PHAGE_Clostr_phiMMP01_NC_028,883 (31)	28.48
*C. difficile* 2,007,855	1	55.9	140	71	1,666,638–1,722,613	PHAGE_Clostr_phiMMP01_NC_028,883 (32)	28.64

### Identification of Antimicrobial Resistance Genes

The AMR analysis from NCBI HPs and RAST yielded different results. From the identified HPs obtained by NCBI, no AMR genes were found by AMRFinderPlus. The analysis of the WGS by AMRFinderPlus revealed the presence of AMR genes *vanZ1* and *blaCDD* which confer resistance to Vancomycin and Beta-Lactam respectively, It also yielded genes with virulence factors *tcdE, tcdB, tcdR,* that were common in all six CD strains. RAST based amino acid sequences revealed that AMR genes *vanZ1*, *blaR1* (Beta-Lactam) and *blaCDD* and genes with virulence factor *tcdE* and *tcdB* were found in all 6 *C. difficile* strains. Additionally, RAST based classified HP amino acid sequences had the virulence variant *tcdR* common in all 6 *C. difficile* strains. ResFinder yielded no AMR genes and virulent factors however in WGS, ResFinder found AMR genes *ant(6)-Ib*, *ant(6)-Ia,* which confer resistance against aminoglycoside and *tet(M)*, *tet(44)* which confer resistance to Tetracycline in *C. difficile* M120 strain and *erm(B)* which confers resistance to Macrolide and *aac(6′)-Im, aph(2″)-Ib* confers resistance to Aminoglycosides in *C. difficile* 2,007,855 (Table 3).

**TABLE 3:** AMR genes determination in WGS strains of *C. difficile* 2,007,855 and M120 using ResFinder tool.

Strain	Resistance Gene	Identity%	Position in Contig	Alignment length	Phenotype	Accession No.
*C. difficile* 2,007,855	*erm(B)*	100	3,136,169..3,136,906	738	Macrolide resistance	U18931
*C. difficile* 2,007,855	*aac(6′)-Im*	96.65	3,810,802..3,811,338	537	Aminoglycoside resistance	AF337947
*C. difficile* 2,007,855	*aph(2″)-Ib*	98.67	3,811,382..3,812,281	900	Aminoglycoside resistance	KF652098
*C. difficile* M120	*ant(6)-Ib*	100	480,747..481,604	858	Aminoglycoside resistance	FN594949
*C. difficile* M120	*ant(6)-Ia*	100	468,126..468,989	864	Aminoglycoside resistance	KF421157
*C. difficile* M120	*tet(M)*	98.85	2,175,639..2,177,558	1920	Tetracycline resistance	EU182585
*C. difficile* M120	*tet(44)*	98.02	478,510..480,432	1923	Tetracycline resistance	NZ_ABDU01000081

### Characterization of Physicochemical Properties

The Instability Index (II), isoelectric point, GRAVY value, and molecular weight of HPs from six strains were determined using the ProtParam tool. In all six strains of *C. difficile*, approximately 70% of the HP sequences had II below 40, indicating that the majority of HPs were stable. *C. difficile* strains R20291 and M120 had more than 280 HP sequences having II values below 40. *C. difficile* strains 196, 2,007,855, and CF5 had approximately 260–275 HP sequences with II values below 40. *C. difficile* strain BR81 had the lowest number of HP sequences, around 240, with an II value below 40. Moreover, HPs from all six strains had theoretical pI ranging from 4.05 to 11.99, while around 70% were found to have negative GRAVY values, indicating that they are non-polar in nature (Supplementary Figure S1). Additionally, detailed information and physicochemical characterization are shown in (Supplementary Sheet S1).

### Identification of Subcellular Localization and Secretory Nature Determination

The subcellular localization of proteins was identified by the PSORTb tool, which classified the HPs from all six strains of *C. difficile* into four categories, namely, cytoplasmic, cytoplasmic membrane, extracellular and unknown, based on their location in the bacterial cell. In all the identified strains of *C. difficile*, approximately 32–38% and 23–27% HPs were localised in the cytoplasm and cytoplasmic membrane, respectively. Meanwhile, 1–3% and 37–40% of all HPs in these six strains were located in the extracellular space, or their location is unknown (Table 4). SignalP 5.0 server was employed to predict the secretory nature of HPs from 6 *C. difficile* strains. Approximately, 87–92% of HPs from each strain were non-secretory, while the remaining proteins were secretory.

**TABLE 4:** Subcellular localisation of *C. difficile* strains hypothetical proteins determined by PSORTb.

Strain	Total HPs	Subcellular Location as Given Be PSORTb			
		Cytoplasmic	Cytoplasmic membrane	Extracellular	Unknown
*C. difficile* BR81	356	122 (34.27%)	86 (24.16%)	10 (2.81%)	138 (38.76%)
*C. difficile* R20291	409	142 (34.72%)	99 (24.21%)	9 (2.20%)	159 (38.88%)
*C. difficile* CF5	413	155 (37.53%)	96 (23.24%)	8 (1.94%)	154 (37.29%)
*C. difficile* M120	404	130 (32.18%)	110 (27.23%)	4 (0.99%)	160 (39.60%)
*C. difficile* 196	374	130 (34.76%)	88 (25.53%)	8 (2.14%)	148 (39.57%)
*C. difficile* 2,007,855	377	133 (35.28%)	90 (23.87%)	9 (2.39%)	145 (38.46%)

### Functional Domain Prediction

Domains are distinct, recurring, functional and structural units of protein, the extent of which can be determined by sequence and structure analysis and are crucial in molecular evolution. Conserved domains contain highly conserved sequence patterns or motifs, which might be detected in a polypeptide sequences. The data obtained from NCBI Batch CDD search tool showed that *C. difficile* BR81, *C. difficile* M120, and *C. difficile* CF5 HPs had nine specific hit types/conserved domains. The functional signature identified in *C. difficile* BR81 strain belongs to nine specific superfamilies namely, Beta_helix, Chalcone_N, GH113_mannanase-like, HDC_protein (x2), M34_PPEP, PBECR3, Pectate_lyase_3, SPASM. Similarly, HPs of *C. difficile* M120 strain had nine specific superfamilies including Beta_helix_3, Chalcone_N, GH113_mannanase-like, HDC_protein (x3), M34_PPEP, PBECR3, SPASM. HPs of *C. difficile* 196 strain had seven specific superfamilies namely Chalcone_N, Glyco_hydro_129, HDC_protein (x2), M34_PPEP, PBECR3, SPASM. Additionally, HPs of *C. difficile* CF5 strain had nine specific superfamilies like ABC_trans_CmpB, C80_toxinA_B-like, Gly_rich, HDC_protein (x3), M34_PPEP, PBECR3, SPASM. Moreover, HPs of *C. difficile* 2,007,855 strain had eight specific superfamilies Chalcone_N, GH113_mannanase-like, Glyco_hydro_129, HDC_protein (x2), M34_PPEP, PBECR3, SPASM. Lastly, HPs of *C. difficile* R20291 strain had eleven specific superfamilies Chalcone_N, DUF5685, DUF5699, DUF5780, GH113_mannanase-like, Glyco_hydro_129, HDC_protein (x2), M34_PPEP, PBECR3, SPASM.

Furthermore, this analysis could be effective in predicting the functional role of HPs determined on the basis of their conserved domains and motifs. Likewise, the common conserved domains identified in HPs of shortlisted strains were HDC_protein, M34_PPEP, PBECR3 and SPASM. The most recurring superfamily Histidine decarboxylase (HDC_protein) is the sole member of the histamine synthesis pathway, producing histamine in a one-step reaction. Histamine cannot be generated by any other known enzyme (Mohammad et al., 2009). M34_PPEP includes the enzyme Pro-Pro endopeptidase (PPEP-1), an extracellular metalloprotease belonging to peptidase family M34. It aids *C. difficile* in switching from an adhesive to a motile phenotype by cleaving cell surface proteins (Lu et al., 2020). PBECR3 (phage-Barnase-EndoU-ColicinE5/D-RelE like nuclease3) is an endoRNase found in polyvalent proteins of phages and conjugative elements (Iyer et al., 2017). SPASM occurs as an additional C-terminal domain in many peptide-modifying enzymes of the radical S-adenosylmethionine (SAM) superfamily (Lu et al., 2020).

### Comparative Homology Analysis

Proteins dissimilar to human proteome are prioritised in therapeutic and vaccine designing, since homologous proteins can cause side effects and cross-reactivity. Those proteins with ≥35% identity, query coverage ≥35%, and E value < 10e-5 were considered. Approximately, 99.7% of the identified HPs across all the selected strains were non-homologous. This indicates that they can be further evaluated for vaccine and other pharmaceutical properties.

### Prediction of Virulence

Virulent proteins assist bacteria in colonising the host and pathogenesis, and these proteins could be cytoplasmic, membranous, or secretory. They help in adhesion, adaptation to the changing environment, and protection against host immune response. Therefore, prioritising these proteins is necessary since they are potential drug targets and immunogenic vaccine candidates. An approximate, 0.25–1.45% of HPs from each strain were found to be virulent in nature, while approximately 99% of proteins from each strain showed no virulence factor.

### Antigenicity Analysis

Antigenicity analysis was determined using the VaxiJen server for 6 *C. difficile* strains. We found that around 39.83% of the *C. difficile* 196 HPs to be antigenic, 40.84% of the *C. difficile* 2,007,855 to be antigenic, 41.01% of *C. difficile* BR81 to be antigenic proteins, 36.07% of *C. difficile* CF5 to be antigenic proteins, 40.09% of the *C. difficile* M120 to be antigenic proteins whereas, 39.85% of the *C. difficile* R20291 to be antigenic proteins. These data suggest that HPs could be further investigated for vaccine properties. Further, we prioritized top antigenic protein from each strain to examine their structure and binding analysis. WP_104,732,835.1 (CD20291strain), WP_009,906,007.1 (CDM120strain), WP_003,429,932.1 (CDCF5strain), WP_021,396,478.1 (CD196strain), WP_021,389,778.1 (CDBR81strain) and WP_003,423,063.1 (CD2007855strain) turned out to be promising candidate and further processed for structure prediction analysis.

### Structure Prediction and Active Site Determination

**FIGURE 3:**
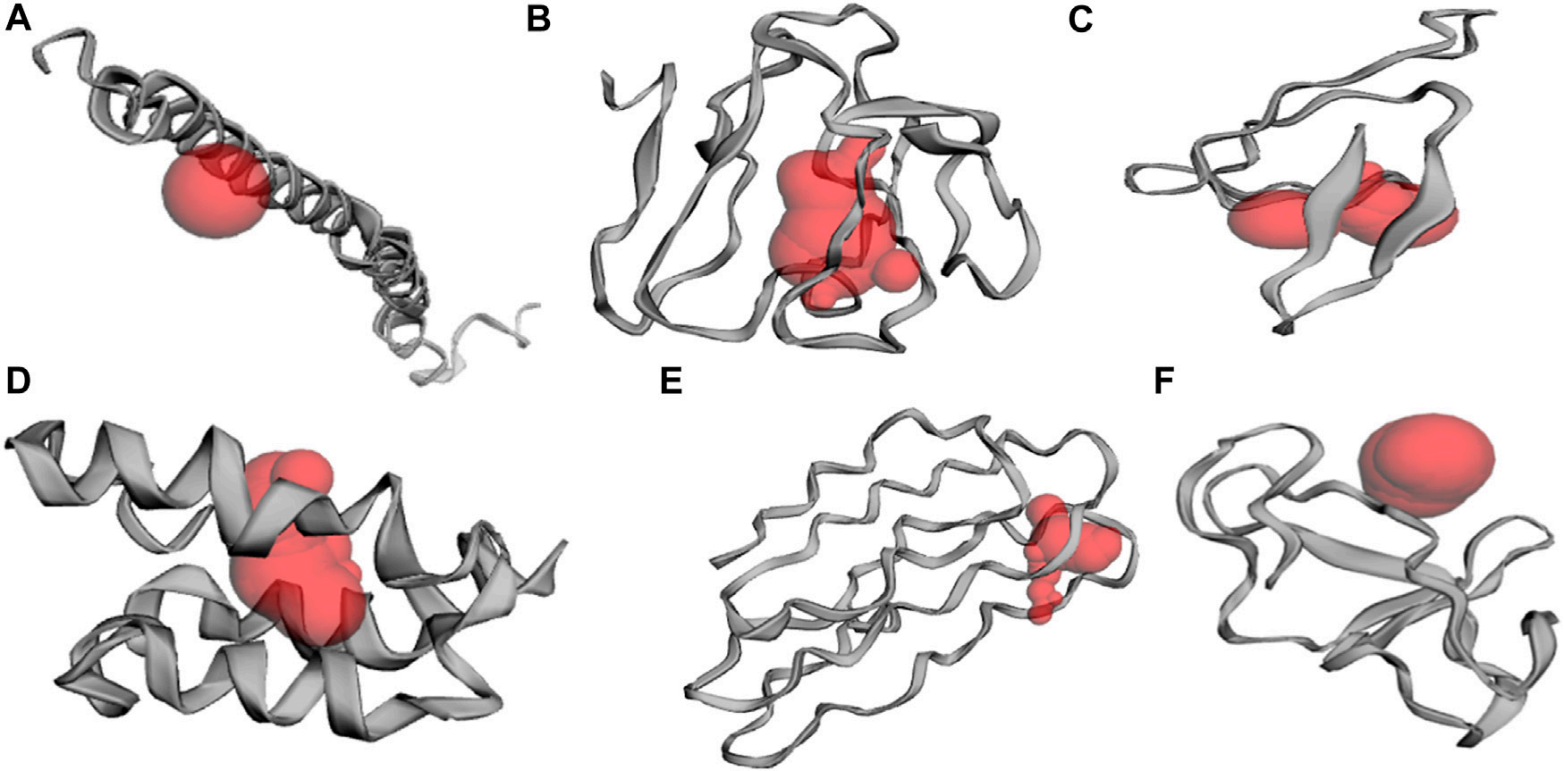
Identification of active site pockets (highlighted in red cavity) for the shortlisted antigenic proteins **(A)** WP_104,732,835.1 **(B)** WP_009,906,007.1 **(C)** WP_003,429,932.1 **(D)** WP_021,396,478.1 **(E)** WP_021,389,778.1, and **(F)** WP_003,423,063.1.

Antigenicity determination allows identification of highly antigenic proteins, which can also assist in filtering out the best potential vaccine candidate (Abbasi et al., 2022). A threshold was selected and six highly antigenic proteins were modelled using the iTASSER server that may be explored for new drug designing strategies. Further, these 3D structural models were subjected to identify active sites by employing CastP server. After pre-processing, the top-ranked potential receptor binding sites and respective residue were identified. All the sites and cavities are shown (Figure 3) and a list of respective residues are provided in Supplementary Table S2.

### Allergenicity Analysis

Therapeutic molecules like vaccines and drugs also have the potential to cause allergic reactions. Therefore, it is essential to check if the protein candidate used acts as an allergen or not. We found that all six *Clostridioides* strains HPs are non-allergens in nature.

## Discussion

Understanding the genomic epidemiology and annotating proteins remains the most impactful strategies to detect, characterise, and monitor pathogens that impact human health. Though traditional biochemical and molecular experiments can be used to assign proper functions for genes, they are expensive, tedious, and have resulted in only 50–60% gene annotations (Sivashankari and Shanmughavel, 2006). Despite continuous research efforts, a large portion of the proteome is still represented by uncharacterized proteins designated as HPs. They are predicted from the nucleic acid sequences and have unknown functions (Varma et al., 2015). Thus, automated gene/protein annotation using bioinformatics tools can overcome this challenge of the post-genome era where complete genome sequences of several organisms are available. Several researchers have gradually worked on structural or functional annotation of HPs from different microbes like *Staphylococcus aureus*, *Vibrio cholera*, *Blumeria graminis* and *Serratia marcescens* and others (Islam et al., 2015; Varma et al., 2015; da Costa et al., 2018; Prabhu et al., 2020). But still, they are shrouded in the mystery and there is a dire need to expedite the process (Omeershffudin and Kumar, 2019). Some of them include deriving information from sequence similarity analysis, interaction of proteins with other proteins or ligands, gene expression profiles, conserved domains/motifs, phylogenetic analysis, phosphorylation regions and active site residue similarity analysis. The most orthodox way of speculating protein function involves sequence similarity analysis using BLAST tool (Varma et al., 2015).

Quite a few studies have elucidated the genomic epidemiology of *C. difficile*, but none of them have focused on the HPs (Cafardi et al., 2013; Ezhilarasan et al., 2013; Basak et al., 2021). Recently, Basak et al., have characterised an *in silico* vaccine using the immunoinformatics approaches via utilising CotE, SlpA and FliC proteins, which were responsible for gastrointestinal tract colonisation, TLR4 interaction, cytokine production, and plays a major role in the adherence of the bacterial cell, which ultimately triggers the innate immune response (Péchiné et al., 2005; Hong et al., 2017; Mori and Takahashi, 2018). Another study has also emphasised on extracellular factors involved in the pathogenesis of *C. difficile*. A unique HP named, CD630_28,300 was found to share sequence similarity with zinc metallopeptidase, which demonstrated the binding of zinc with CD630_28,300 and its ability to disrupt the human fibronectin network by cleaving the fibronectin and fibrinogen *in vitro* in a zinc-dependent manner (Cafardi et al., 2013). Researchers have also employed similarity searches between pathogen and host, essentiality analysis, metabolic functional association, and choke point analysis. They identified 19 promising drug targets which were non-homologous to host proteins, and participated in four pathogen specific pathways, of which the peptidoglycan biosynthesis was found to be the highest contributor to the list of potential target proteins. MurG enzyme from the peptidoglycan biosynthesis pathway was found as one of the potential targets (Ezhilarasan et al., 2013).

In this study, the *C. difficile* HPs identified from NCBI database were subjected to various *in silico* experiments like, physicochemical properties, subcellular localisation identification, transmembrane helices detection, comparative homology analysis, antigenicity analysis, allergenicity analysis, secretory nature detection, and AMR identification. Furthermore, the top antigenic HPs were shortlisted and subjected to structure prediction and binding site analysis. The analysis of six *Clostriodioles* strains resulted in identifying approximately 11% of HPs from around 3,500 proteins coded by nearly 4.1 Mb genome size of each strain. The physicochemical properties of HP from 6 *C. difficile* strains, namely*,* BR81, R20291, CF5, M120, 196, and 2,007,855, were analysed. In all these strains of *C. difficile*, approximately 70% of the HPs were found to be stable as they had instability index (II) value below 40. Isoelectric point (pI) and grand average of hydropathicity (GRAVY) value were other important physicochemical parameters that were determined. The pI of HP ranges from 4.05 to 11.99 in all the strains. Isoelectric point (pI) is that pH where the amino acid of protein has a net zero charge and hence does not move in a direct current electrical field. At pI solubility of protein is lowest and electro focussing system mobility is zero, thereby making proteins stable and compact at this pH. This information can be utilised to develop buffer system for protein purification by isoelectric focussing (Islam et al., 2015). The GRAVY number of proteins is the measure of its hydrophilicity or hydrophobicity which are combined in a hydropathy scale. A positive value indicates proteins are hydrophobic while a negative value indicates that they are hydrophilic (Chang and Yang, 2013). In present study, around 70% HP of these strains had negative GRAVY values, demonstrating that they are hydrophilic in nature.

Since proteins located on the cell membrane can act as potential vaccine targets and those in the cytoplasmic matrix can act as potential drug targets, therefore, knowledge from subcellular localization is an important parameter for functional characterization of a protein (Prabhu et al., 2020). Moreover, research suggests the role of cell surface proteins in Clostridial pathogenesis, yet not many cell surface or secreted proteins of the nosocomial pathogen *C. difficile* have been identified or functionally characterised (Cafardi et al., 2013). Protein subcellular localization of HPs from all six strains of *C. difficile* were examined by PSORTb tool which categorises them into four categories, namely, cytoplasmic, cytoplasmic membrane, extracellular and unknown, based on their location in the bacterial cell. Approximately, 32–38% and 23–27% HPs were localised in the cytoplasm and cytoplasmic membrane, respectively. Meanwhile, 1–3% and 37–40% of all HPs in these six strains were located in the extracellular space, or their location was unknown.

Prediction of signal peptides is a key feature to determine the transportation system of particular proteins and their cleavage site. All non-cytoplasmic proteins have signal peptides that facilitate the transport of proteins across the membrane to a designated cellular location or organelles (Prabhu et al., 2020). We have used SignalP 5.0 server and found that almost 87–92% of HPs from each strain did not have signal peptides while the remaining 8–13% proteins had signal peptides indicating their involvement in a secretory pathway. Membrane proteins are also involved in various biological processes like signalling, transport, energy transduction and pathogenesis and can act as potential drug targets. Thus, it is important to predict membrane proteins to develop potent drug molecules (Prabhu et al., 2020).

Consequently, to check whether these HPs *C. difficile* can act as potential vaccine targets, we evaluated their sequence homology with humans, virulence factor, antigenicity and allergenicity. For a protein to be considered a potential candidate, it should be non-homologous to human proteins to avoid cross-reactivity with them. Also, they should be antigenic, non-allergenic and can have presence of virulence factors, our study demonstrated that almost 99.7% HP from all strains were non homologous but only a minute fraction of them around 0.25–1.45% has virulent factor. While around 36–41% of all HP were antigenic and none of them were allergens. Additionally, AMR analysis identified that the glycopeptide resistance protein *vanZ1* gene, glycosylating toxin *TcdB* gene, holin-like glycosylating toxin export protein *TcdE*, glycosylating toxin sigma factor *TcdR*, and CDD family class D beta-lactamase *blaCDD* genes were common in all strains according to the AMR and virulent factor data generated by AMRFinderPlus for WGS sequences, of which the *vanZ1* and the *blaCDD* genes were AMR, rest of the above mentioned genes were virulent. The AMR genes identified by ResFinder in only the WGS strains of M120 and 2,007,855 were also identified as AMR genes by the AMRFinderPlus. In the protein sequence from RAST three genes *vanZ1*, *tcdB*, and *blaR1* were found in all six chains, of which the *tcdB* is a virulence factor. In the HPs from RAST, the AMRFinderPlus was able to identify genes with virulence factors and the *tcdR* gene was the common gene in all six chains. The HPs play a role in virulence and could be used as potential targets for drug discovery or as antigen to develop vaccines. These properties of virulence, stability, polarity, presence in cytoplasmic and extracellular regions, minimal number of transmembrane helices present, non-homology with the human proteome, antigenicity and non-allergenicity which these HPs show, further experimental and computational studies can be done to assess the potentiality of these HPs as targets for drug discovery and as vaccine candidates.

## Conclusion

Identifying protein functions is crucial for understanding various biological processes. Here, we implemented *in silico* approaches to predict the function of HPs from six strains of the *C*. *difficile.* While employing various tools to annotate and characterise HPs, characteristic predictions like subcellular localization, secretory nature and physicochemical properties were suitable to understand particular features. Further, identification of AMR genes, prophage sequences, CRISPR-Cas9 genes, and elucidation of immunoinformatics properties has allowed us to identify proteins that might play important roles in the complex mechanisms and biological processes. In conclusion, the pipeline used in this study allowed us to screen and characterise candidate HPs for assigning protein functions and further broaden up the possibilities for better downstream validation.

## Data Availability

The original contributions presented in the study are included in the article/Supplementary Material, further inquiries can be directed to the corresponding authors.

## Abbreviations

C. difficile, *Clostridium difficile*; CDI, *C. difficile* infection; CDD, Conserved Domain Search; AMR, Antimicrobial resistance; II, Instability Index; GRAVY, Grand Average of Hydropathicity Value; CRISPR, Clustered regularly interspaced short palindromic repeats; Cas9, CRISPR associated protein 9; HP, Hypothetical Protein; NCBI, National Centre for Biotechnology Information; PHASTER, PHAge Search Tool Enhanced Release.
