# Supplementary figures and images for: In Silico Characterization of Uncharacterized Proteins From Multiple Strains of Clostridium Difficile
Source: Front Genet. 2022 Aug 11;13:878012. doi: 10.3389/fgene.2022.878012 (PMC9403866; doi:10.3389/fgene.2022.878012)

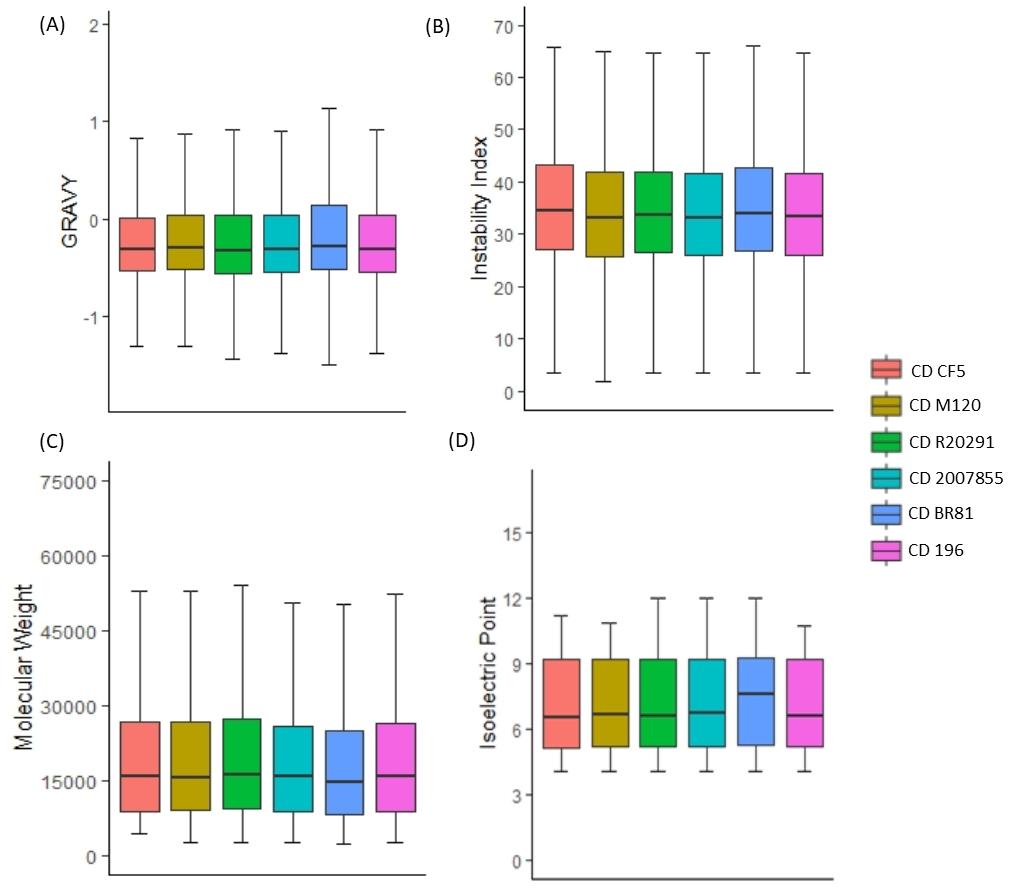

Supplement: Supplementary file 2 [file Image1.JPEG]
