# Supplementary material for: In Silico Characterization of Uncharacterized Proteins From Multiple Strains of Clostridium Difficile
Source: Front Genet. 2022 Aug 11;13:878012. doi: 10.3389/fgene.2022.878012 (PMC9403866; doi:10.3389/fgene.2022.878012)
Supplement: Supplementary file 3 [file DataSheet1.docx]

**
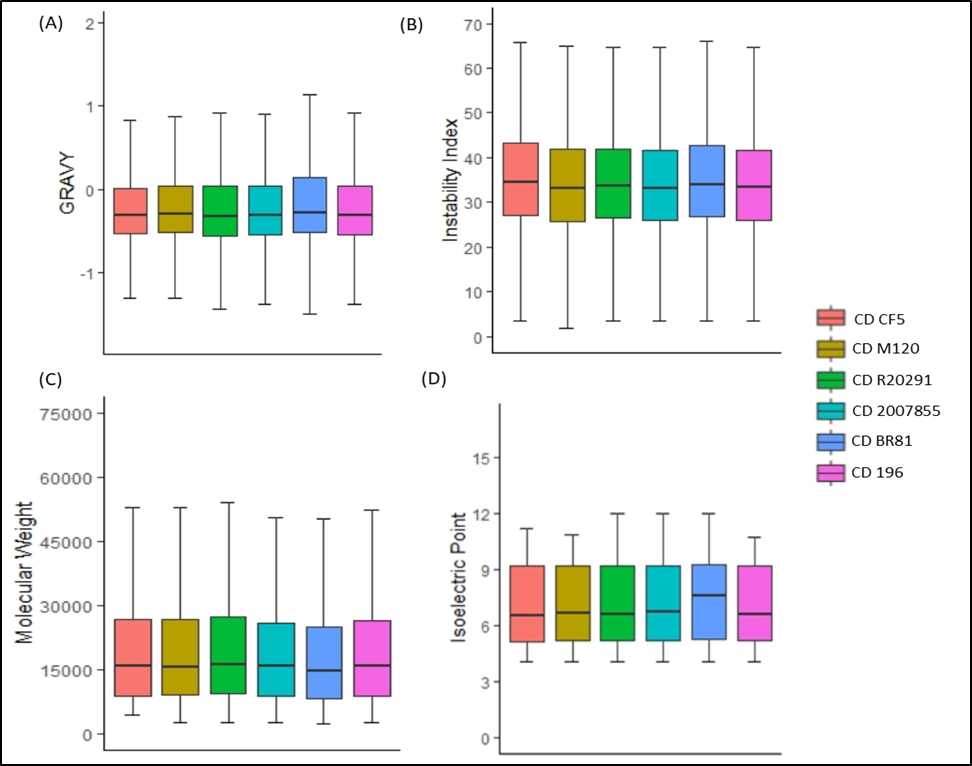
**

**Supplementary FIGURE 1**. Whisker plots representing the physicochemical properties of the shortlisted *Clostridium* d*ifficile* strains hypothetical proteins. (A) Grand Average Hydropathicity Value (B) Instability Index (C) Molecular Weight (D) Isoelectric point.

**Supplementary Table 1: Characterization of CRISPR loci and Cas genes in *Clostridium Difficile* strains.**

| **Strain** | **CRISPR_Start** | **CRISPR_End** | **CRISPR_Length** | **Spacers_Nb** | **Evidence_Level** |
| --- | --- | --- | --- | --- | --- |
| *Clostridioides difficile*_2007855 | 1288149 | 1289102 | 953 | 14 | 4 |
| *Clostridioides difficile*_2007855 | 1497487 | 1498239 | 752 | 11 | 4 |
| *Clostridioides difficile*_2007855 | 1608857 | 1609150 | 293 | 4 | 4 |
| *Clostridioides difficile*_2007855 | 1841322 | 1842537 | 1215 | 18 | 4 |
| *Clostridioides difficile*_2007855 | 2191897 | 2192522 | 625 | 9 | 4 |
| *Clostridioides difficile*_2007855 | 3263982 | 3265595 | 1613 | 24 | 4 |
| *Clostridioides difficile*_2007855 | 3510931 | 3511553 | 622 | 9 | 4 |
| *Clostridioides difficile*_BR81 | 752098 | 753834 | 1736 | 26 | 4 |
| *Clostridioides difficile*_BR81 | 1257179 | 1257736 | 557 | 8 | 4 |
| *Clostridioides difficile*_BR81 | 1500847 | 1501926 | 1079 | 16 | 4 |
| *Clostridioides difficile*_BR81 | 1851001 | 1852212 | 1211 | 18 | 4 |
| *Clostridioides difficile*_BR81 | 2226341 | 2227556 | 1215 | 18 | 4 |
| *Clostridioides difficile*_ BR81 | 2402960 | 2404049 | 1089 | 16 | 4 |
| *Clostridioides difficile*_BR81 | 2514308 | 2514668 | 360 | 5 | 4 |
| *Clostridioides difficile*_BR81 | 2722783 | 2723141 | 358 | 5 | 4 |
| *Clostridioides difficile*_CD196 | 1294727 | 1295680 | 953 | 14 | 4 |
| *Clostridioides difficile*_CD196 | 1504069 | 1504821 | 752 | 11 | 4 |
| *Clostridioides difficile*_CD196 | 1615439 | 1615732 | 293 | 4 | 4 |
| *Clostridioides difficile*_CD196 | 1849663 | 1850878 | 1215 | 18 | 4 |
| *Clostridioides difficile*_CD196 | 2200244 | 2200869 | 625 | 9 | 4 |
| *Clostridioides difficile*_CD196 | 3244496 | 3246042 | 1546 | 23 | 4 |
| *Clostridioides difficile*_CD196 | 3491430 | 3492052 | 622 | 9 | 4 |
| *Clostridioides difficile*_CF5 | 1649491 | 1650177 | 686 | 10 | 4 |
| *Clostridioides difficile*_CF5 | 1741664 | 1742176 | 512 | 7 | 4 |
| *Clostridioides difficile*_CF5 | 1742778 | 1743729 | 951 | 14 | 4 |
| *Clostridioides difficile*_CF5 | 1887965 | 1889572 | 1607 | 24 | 4 |
| Clostridium_difficile _M120 | 1343256 | 1343882 | 626 | 9 | 4 |
| Clostridium_difficile _M120 | 1826292 | 1827440 | 1148 | 17 | 4 |
| Clostridium_difficile _ M120 | 2152896 | 2155826 | 2930 | 44 | 4 |
| Clostridium_difficile_M120 | 2220611 | 2221499 | 888 | 13 | 4 |
| Clostridium_difficile_M120 | 2331600 | 2334132 | 2532 | 38 | 4 |
| Clostridium_difficile _M120 | 3266657 | 3268778 | 2121 | 32 | 4 |
| *Clostridioides difficile*_R20291 | 1305910 | 1306867 | 957 | 14 | 4 |
| *Clostridioides difficile*_R20291 | 1515256 | 1516008 | 752 | 11 | 4 |
| *Clostridioides difficile*_R20291 | 1626627 | 1626920 | 293 | 4 | 4 |
| *Clostridioides difficile*_R20291 | 1859093 | 1860047 | 954 | 14 | 4 |
| *Clostridioides difficile*_R20291 | 2294363 | 2294988 | 625 | 9 | 4 |
| *Clostridioides difficile*_R20291 | 3338591 | 3340337 | 1746 | 26 | 4 |
| *Clostridioides difficile*_R20291 | 3585725 | 3586347 | 622 | 9 | 4 |

**Supplementary Table 2: Identification of active amino acid residues in the shortlisted proteins for *Clostridium difficile* strains.**

| Strain | Protein | Active Amino Acid Residues |
| --- | --- | --- |
| CDR20291 | WP_104732835.1 | ARG62, ALA65, ARG66 |
| M120 | WP_009906007.1 | HIS37, HIS37, ASP56, HIS57, HIS59, ASP61, PHE63, THR66, HIS67, GLU68, HIS69, SER70, GLU72, HIS73,HIS89, LYS90 |
| CF5 | WP_003429932.1 | ILE8, ILE10, ILE15, LYS16, VAL17, GLU18, PHE19, ASN20, ARG21, VAL26, ASP27, ILE28, LYS29, VAL30, ARG31, VAL47 |
| CD196 | WP_021396478.1 | MET1, LEU2, ALA14, LYS15, PHE17, TYR18, ASN21, GLU22, ILE25, LEU30, CYS37, LEU38, ALA41, TYR45, PHE51, VAL55, VAL59, ILE62, TRP66 |
| CDBR81 | WP_021389778.1 | CYS19, CYS20, THR22, GLY24, GLU25, GLY26, CYS27, CYS28, ASN51, GLY52, VAL53, GLY54, HIS57, HIS59 |
| CD2007855 | WP_003423063.1 | ARG6, GLN22, TYR24, GLU51, VAL52 |
